# Editorial: Bioscience of D-amino Acid Oxidase From Biochemistry to Pathophysiology

**DOI:** 10.3389/fmolb.2018.00108

**Published:** 2018-11-28

**Authors:** Loredano Pollegioni, Jumpei Sasabe

**Affiliations:** ^1^Dipartimento di Biotecnologie e Scienze della Vita, Università degli Studi dell'Insubria, Varese, Italy; ^2^Department of Pharmacology, Keio University School of Medicine, Tokyo, Japan

**Keywords:** D-amino acid, NMDA receptor, D-amino acid oxidase, enzyme regulation, brain

We all now know that homochirality is biologically highly relevant: a number of biomolecules contain chiral centers and this determines the spatial architecture of biopolymers, the recognition selectivity, and the biological role. All α-amino acids, with the sole exception of glycine, possess a chiral center (i.e., the α-C) resulting in two “mirror” molecules, namely, L- and D-amino acids. Despite identical physical and chemical properties, only the L-enantiomers were selected during evolution as protein constituents (Lamzin et al., 1995). The biological relevance of D-amino acids in mammals started to emerge in the 1990s, when novel sensitive analytical techniques (reviewed at the website www.d-aminoacids.com) made it possible to separate and quantify amino acid enantiomers (Hashimoto et al., 1992). Now we know that D-aspartate and D-serine occur intrinsically in mammals and show spatiotemporal alterations in the central nervous system, which shapes various neurobiological processes that are distinct from those of their L-counterparts. Indeed, D-aspartate and D-serine (as well as the achiral glycine) were demonstrated to act as N-methyl-D-aspartate (NMDA) receptor ligands at different binding sites, thus regulating the receptor functionality (Mothet et al., 2000; Errico et al., 2008; Billard, 2012). NMDA receptors mediate excitatory neurotransmission, and optimal activation of the receptors is crucial for neuronal homeostasis, while altered NMDA receptor activity is implicated in multiple pathological conditions.

In 1999, the mouse enzyme responsible for D-serine synthesis in the brain was identified (Wolosker et al., 1999), opening the investigation of D-amino acid metabolism in mammals (Pollegioni and Sacchi, 2010). Accordingly, it is essential to define the properties of the enzymes involved in the synthesis and degradation and to determine the significance for brain physiological processes and relevant pathological conditions, such as schizophrenia, Alzheimer's disease, neuropathic pain, amyotrophic lateral sclerosis (ALS), depression, infections, etc.

This special issue focuses on the peculiar properties of the main mammalian enzyme involved in D-serine metabolism, namely, D-amino acid oxidase (DAAO, EC 1.4.3.3). Sacchi et al. have reviewed present knowledge of human DAAO (hDAAO) and Murtas et al. add further information related to its substrate specificity, showing that D-cysteine is the best substrate and proposing it as a putative physiological substrate in selected tissues (e.g., gut). hDAAO shows a preference for hydrophobic amino acids, such as D-kynurenine, D-DOPA, and D-tryptophan, molecules which are relevant in neurotransmission. This experimental evidence indicates that hDAAO plays tissue-specific roles. The review by Koga et al., reporting on the distribution of DAAO in mouse tissues, also focuses on the wide substrate preference of the enzyme in rodents and on the accumulation of selected D-amino acids in tissues and body fluids of mice expressing an inactive form of the enzyme. The main physiological substrates of mouse DAAO were identified as D-alanine (generated from bacteria and associated with reactive oxygen species generation) and D-serine (produced by serine racemase and acting as a neuromodulator).

Notably, hDAAO possesses a very low affinity for the FAD cofactor (Caldinelli et al., 2009) and thus it is present in solution largely as an inactive apoprotein form. Murtas et al. demonstrate that this latter species is present in two conformations differing in flavin affinity: the higher-affinity conformation is generated when a ligand (such as the substrate or a competitive inhibitor) is bound in the active site.

A further mechanism of hDAAO regulation is due to interaction with pLG72, a small (153 amino acids) primate-specific protein. The review by Pollegioni et al. presents the roles that have been proposed for pLG72 in recent years, highlights the controversy over pLG72 expression in human tissues, and focuses on the molecular details of the interaction with hDAAO, the consequences of binding on its function and stability and on D-serine cellular levels. Details about the main effects related to the pathological R30K substitution in pLG72, corresponding to the SNP rs2391191 genetically linked to schizophrenia (Chumakov et al., 2002), are also reported. The definition of the molecular details of the hDAAO-pLG72 complex now offers the opportunity to modulate NMDA receptor function acting at the D-serine cellular level. This can be achieved by designing molecules able to modulate the hDAAO-pLG72 interaction or, in a most classical way, to inhibit hDAAO. The insightful review by Molla reports on the studies performed in the past 10 years at a number of academic and industrial laboratories which aimed to identify effective hDAAO inhibitors to restore the physiological concentration of D-serine. Although molecules with at least three different mechanisms have been reported, none of the identified compounds has reached the market yet.

Sacchi et al. and Kondori et al. report on the potential significance of hDAAO variants in human pathological states. These studies offer the reader deep insight into the structure-function relationships of this flavoenzyme and a functional classification of hDAAO variants based on enzymatic activity, i.e., hypo- and hyperactive enzyme forms. Kondori et al. have focused on the R199W pathogenic substitution in hDAAO associated with a family line of autosomal dominantly inherited ALS, resulting in altered D-serine levels and causing protein aggregation, autophagy, and cell death in primary cultures of motor neurons. The authors report about the attenuated generation of protein ubiquitinated inclusions, the increase in autophagosome production, and reduced apoptotic cell death by a selective inhibitor of the co-agonist binding site of the NMDA receptor. A transgenic mice line expressing the enzyme variant with the pathogenic mutation is also presented: notably, heterozygous expression of the R199W hDAAO variant led to a significant loss of spinal cord motor neurons at 14 months.

Finally, a useful and detailed description of the main assays for detecting DAAO activity has been provided by Rosini et al. It is interesting to note the diversity of assays that can be used to determine DAAO activity on different samples, from recombinant proteins to cells and tissue samples. These direct and indirect methods are based on the detection of dioxygen consumption, the reduction of alternative electron acceptors, or the generation of α-keto acids.

Present knowledge indicates that the structural-functional properties of hDAAO seem to have evolved in order to generate a generalist enzyme characterized by a broad substrate acceptance and by different regulation mechanisms that make it possible to fine tune its activity and stability. In such a way, hDAAO is able to meet different physiological needs related to D-amino acid metabolism in different tissues.

## Author contributions

All authors listed have made a substantial, direct and intellectual contribution to the work, and approved it for publication.

### Conflict of interest statement

The authors declare that the research was conducted in the absence of any commercial or financial relationships that could be construed as a potential conflict of interest.
